# The Potential of Artificial Intelligence Tools for Reducing Uncertainty in Medicine and Directions for Medical Education

**DOI:** 10.2196/51446

**Published:** 2024-11-04

**Authors:** Sauliha Rabia Alli, Soaad Qahhār Hossain, Sunit Das, Ross Upshur

**Affiliations:** 1 Temerty Faculty of Medicine, University of Toronto, Toronto, ON, Canada; 2 Department of Computer Science, Temerty Centre for AI Research and Education in Medicine, University of Toronto, 27 King's College Circle, Toronto, ON, M5S 1A1, Canada, 1 6478922470; 3 Intermedia.net Inc., Sunnyvale, CA, United States; 4 Department of Surgery, University of Toronto, Toronto, ON, Canada; 5 Keenan Chair in Surgery, Division of Neurosurgery, St. Michael's Hospital, Toronto, ON, Canada; 6 Dalla Lana School of Public Health, Department of Family and Community Medicine, University of Toronto, Toronto, ON, Canada

**Keywords:** artificial intelligence, machine learning, uncertainty, clinical decision-making, medical education, generative AI, generative artificial intelligence

## Abstract

In the field of medicine, uncertainty is inherent. Physicians are asked to make decisions on a daily basis without complete certainty, whether it is in understanding the patient’s problem, performing the physical examination, interpreting the findings of diagnostic tests, or proposing a management plan. The reasons for this uncertainty are widespread, including the lack of knowledge about the patient, individual physician limitations, and the limited predictive power of objective diagnostic tools. This uncertainty poses significant problems in providing competent patient care. Research efforts and teaching are attempts to reduce uncertainty that have now become inherent to medicine. Despite this, uncertainty is rampant. Artificial intelligence (AI) tools, which are being rapidly developed and integrated into practice, may change the way we navigate uncertainty. In their strongest forms, AI tools may have the ability to improve data collection on diseases, patient beliefs, values, and preferences, thereby allowing more time for physician-patient communication. By using methods not previously considered, these tools hold the potential to reduce the uncertainty in medicine, such as those arising due to the lack of clinical information and provider skill and bias. Despite this possibility, there has been considerable resistance to the implementation of AI tools in medical practice. In this viewpoint article, we discuss the impact of AI on medical uncertainty and discuss practical approaches to teaching the use of AI tools in medical schools and residency training programs, including AI ethics, real-world skills, and technological aptitude.

## Introduction

In clinical practice, uncertainty refers to a physician’s perceived inability to accurately explain or advise on a patient’s medical problem [1] and may arise at any stage of the patient encounter, be it assessment, investigation, diagnosis, or treatment [2]. Physicians have both a professional and instinctive desire to be as certain as possible when diagnosing or treating patients [3]. While teaching physicians to tolerate uncertainty is important, there is also a need to overcome problems in medicine that contribute to uncertainty in the physician’s mind. Several models of uncertainty have been proposed, but for the ease of our discussion, the distinction between reducible and irreducible uncertainty is most relevant.

Simply put, uncertainties associated with unknowable things are irreducible, and uncertainties associated with knowable things that are currently unknown are reducible [4]. Reducible forms of uncertainty in medicine may stem from the lack of information about the effects of treatment; information overload or complexity; vagueness of terms; or differing beliefs, values, and preferences among providers [5]. Reducible uncertainty can be overcome by obtaining new knowledge. For example, an 85-year-old woman with a headache and a pulsatile temporal artery with a normal erythrocyte sedimentation rate and normal C-reactive protein (CRP) levels may be treated for giant cell arteritis (GCA) with pulse steroids in the emergency department by one provider, but might be deemed unlikely to have the same diagnosis by another. The uncertainty may arise, in this case, from any number of factors, including the lack of a consensus definition of GCA, differing tolerances of risk among providers, and the overall clinical appearance of the patient.

Irreducible forms of uncertainty in medicine, on the other hand, stem from statistical limitations (eg, random error due to natural variation), epistemic problems (such as measurement error, systematic error, model uncertainty, and uncertainty about inducing case probability from class probability), or numerical vagueness [5]. For example, again considering the diagnosis of GCA, both an elevated erythrocyte sedimentation rate and elevated CRP level (eg, CRP level >10 mg/dL) may help to confirm the diagnosis. However, even a CRP level of 12 mg/dL may not be considered elevated for older age, obesity, and smoking (all of which are factors that may raise the CRP level). The uncertainty in this case is due to the difficulty in applying the broader diagnostic criteria to the specific patient being assessed, and natural variation in numerical criteria used to make the diagnosis. Even with the development of more specific numerical cutoffs for CRP levels, patients with levels slightly above the threshold may not truly have an increased risk of the disease. These are foundational problems of knowledge creation and not specific to medicine. As such, irreducible forms of uncertainty cannot be eliminated by obtaining new knowledge.

Artificial intelligence (AI) tools are being increasingly used as adjuncts to improve diagnosis, medical decision-making, and treatment. Here, the distinction between reducible and irreducible uncertainty becomes important; the forms of uncertainty that can be improved by obtaining knowledge (namely, the reducible forms) may see a benefit with the introduction of AI tools in medical practice. AI tools that have been developed can assist physicians with documenting encounters [6], diagnosing skin cancer [7], providing patient information on medical conditions [8], and teaching surgical skills to medical trainees [9].

Despite the promise AI tools may hold to overcome the sources of uncertainty in medicine, the relationship between AI and medical uncertainty has not been explored in the literature. In this viewpoint article, we consider the potential of AI tools to reduce uncertainty in medical practice, when used as adjuncts to clinical reasoning. In addition, we offer practical approaches to teaching the use of AI in medical schools and residency programs to increase the uptake of these tools in practice.

## Impact of AI on Reducible Uncertainty

The potential impact of AI tools on reducible uncertainty in medical practice is vast. We will, however, focus our discussion on three sources of reducible uncertainty: (1) lack of clinical information, (2) provider competence, and (3) provider bias.

### Clinical Information

A significant source of uncertainty in medical practice is the lack of availability of relevant information to make a decision. This may include the lack of studies about the disease process or the lack of information about the particular patient. AI tools are currently being employed to gather scientific data, through large database management and integration with biobanks [10], and integrate these biological and clinical variables in prediction outputs [11]. This accelerated pace of data gathering may substantially advance our understanding of disease. In addition to the limited understanding of disease in the scientific community, the lack of information about the particular patient can also create uncertainty. Consider a patient who presents for psychiatric assessment with suicidal ideation. A detailed history is required to arrive at the underlying cause of distress, but it is time-consuming to elicit this information and is affected greatly by patient rapport. AI tools, including scribes [12], can help to address such time constraints, by reducing time spent on documentation and administrative tasks. AI may also provide feedback on a physician’s skills in providing patient-centered care, facilitating improvement in this domain [13]. In addition, patients may be more willing to provide socially negative information to AI programs than to physicians, assisting with the collection of data used in clinical decision-making [14].

### Provider Competence

Provider skill, knowledge, and experience may also lead to diagnostic uncertainty. AI tools are currently being developed as clinical decision support aids. For example, deep neural networks have been able to classify skin cancer from dermoscopic images at similar levels of accuracy to board-certified dermatologists [15]. Similarly, AI tools have been developed to support the triage process in emergency departments with a 27% greater accuracy than that of the average nurse [16]. These tools have the tremendous potential of reducing human error and contributing to personal learning and process improvement.

### Provider Bias

Differing beliefs, values, and preferences among physicians also contribute to reducible uncertainty in diagnosis and treatment. Medical decision-making is ideally a balance of the best available evidence and clinical gestalt, the latter being influenced by unconscious biases. For example, in cases where the incidence of disease is lower in a particular population, a reliance on heuristics may result in underdiagnosis. Take for example, the diagnosis of cutaneous malignant melanoma, which has a lower incidence rate in people with darker skin color compared to non-Hispanic individuals with lighter skin color [17]. Research has shown that patients with darker skin types are more likely to present with later stage cancers [18], resulting in higher mortality rates [19]. AI could assist with addressing disagreements by providing recommendations, irrespective of the decision-making agent’s personal perspectives, beliefs, or biases. In fact, a recent study demonstrated that ChatGPT could predict dermatoses in people with lighter and darker skin color with similar levels of accuracy [20], despite established clinical disparities in the diagnosis of skin conditions between these groups [21].

## Impact of AI on Irreducible Uncertainty

Despite the potential of AI tools to affect the reducible forms of uncertainty, there are irreducible forms of uncertainty in medicine that these tools will not resolve. Here, we will discuss two irreducible forms of uncertainty, (1) the application of class-to-case probability and (2) model uncertainty, and how AI tools will impact them.

### Class-to-Case Probability

The distinction between class probability and case probability as a source of uncertainty was first described by Austrian economist and philosopher, Ludwig von Mises [22]; he described class probability as a general understanding of risk for a particular group of people, and case probability as the specific understanding of risk for an individual. For example, based on large population studies, we understand that by the age of 80 years, 14% of smokers will develop lung cancer [23]. However, for a particular 65-year-old smoker who comes into the office, whether they will develop lung cancer remains uncertain. Their risk is based on several immeasurable factors, including their comorbidities, environmental exposures, and genetic profile. Regardless, medical decision-making routinely involves an abstraction of class probability to case probability, and we reasonably accept this patient’s risk of lung cancer to be 14%.

What is the impact of AI on this problem? AI tools are capable of analyzing large datasets and identifying patterns that may improve the overall accuracy in estimating the risk for groups of patients (class probabilities). Additionally, these tools are being increasingly used to advance the field of precision medicine [24]. For example, genotype-guided treatment is an area of active research in precision medicine. Genomic profiling can be used to provide targeted therapy for patients with lung or breast cancer [25]. By integrating massive amounts of individual data (genetics, lifestyle, and environmental exposure), AI may be able to better predict how a specific patient might respond to treatment, improving our understanding of case probability. Additionally, these tools are also able to learn continuously and may be able to refine their predictions regarding disease risk and prognosis as more information becomes available. Wearable devices, for example, which collect continuous, multidimensional data during daily activities, have captured subtle changes in cognition and functional capacity long before the onset of dementia [26]. Despite these advances, decision-making in medicine will continue to rely on the abstraction of class probability to case probability, as the future outcome of a particular case can never be predicted with complete certainty. This is an epistemological source of uncertainty that AI may be able to mitigate, but never eliminate.

### Model Uncertainty

Model uncertainty is another reducible form of uncertainty in medicine. This form of uncertainty arises from the fact that models of disease are approximations of complex systems and involve simplifications of reality and assumptions. As a result, these models may not explain all presentations of a disease. For example, we understand a depletion in serotonin as being a cause of depression [27]; however, this model is imperfect and does not explain why some people who meet the criteria for depression do not respond to selective serotonin reuptake inhibitors. One advantage AI tools offer is that they are data-driven rather than assumption-driven. Deep learning techniques can allow AI tools to learn from raw data rather than predefined model parameters. For example, AI tools used in the COVID-19 pandemic were able to identify unusual cases of pneumonia before public health authorities recognized the threat [28]. Unlike traditional models that are fixed once developed, AI models can also learn and adapt over time as new data becomes available. This means that AI tools can develop models that change over time, at rates much faster than traditional scientific models were developed.

While these tools may reduce model uncertainty, one limitation of AI tools when used to develop models of disease is the lack of explainability or algorithmic transparency. It is not always easy or possible to understand how and why an AI system arrives at its decision [29]. Currently, the tools, methods, policies, and frameworks required for explainable AI have not been well developed [11]. This lack of transparency may increase model uncertainty, due to a lack of physician trust in the model’s decision. While explainable AI is foreseeable with time and technological advances, on an epistemological level, AI cannot overcome the fundamental limitation that models are approximations of reality with inherent error. Therefore, model uncertainty will persist as a challenge to medical decision-making despite advances in AI.

## Teaching AI to Medical Learners

### Overview

Despite the promise AI tools hold in reducing uncertainty, there has been considerable resistance to the implementation of AI tools in medical practice. Several reasons for this reluctance exist, including a lack of transparency, cost, privacy issues, reputation concerns, and legal liability [30]. Some medical professionals also perceive AI systems as threats to medical professional identity (recognition and capability) [31]. In addition, patients worry that these tools may not be able to account for their unique preferences the way a physician might [32]. These concerns are valid and will need to be addressed before AI tools reach widespread implementation. Indeed, our understanding of AI ethics and privacy issues has greatly improved in the last 5 years [33]. Despite these barriers, AI tools have already made their way into medical practice. In fact, learners are increasingly voicing an interest in training on how to use AI technologies in medical practice [34]. Consequently, a shift in perspective is required in medical education to teach learners how to practically use these tools and to understand their benefits and limitations.

Novel teaching approaches are needed to train medical learners to use AI tools practically and responsibly. For educators involved in designing medical curricula, three objectives should be considered: (1) improving students’ understanding of capabilities, limitations, and ethics of AI use; (2) increasing practical skills in the use of AI; and (3) increasing technological aptitude needed for producing AI systems.

### Teaching the Capabilities, Limitations, and Ethics of AI Use

Students should receive formal education on the capabilities and limitations of AI tools. This includes the scope of technologies presently available in various fields of medicine, in addition to those that are newly emerging, including AI scribes, triage assistants, and patient-facing chatbots. Learners should also be made aware of the limitations of these technologies, including the potential for error due to assumptions of class-to-case probability and model uncertainty. Generative forms of AI, including ChatGPT, experience the lack of context and generalizability and are consequently at risk of spreading misinformation [35]. This phenomenon, known as “hallucination,” refers to the generation of information that appears statically plausible but may not be accurate [36]. Students must be made aware of these limitations. In addition, students should be educated about the ethical and legal risks and issues, misinformation, and hallucinations. This includes social discrimination and racial bias in datasets used to develop these tools, which may be further perpetuated by these tools [37]. The legal implications of AI use should also be discussed. While the legalities around AI use are currently being debated, there is a possibility of medical negligence and liability to both physicians and medical institutions if undue harm to the patient is caused by these tools [38]. Students should also be informed about the privacy and security considerations of sharing personal health information with AI tools, including generative forms of AI, such as ChatGPT [39,40]. The capacity of AI tools to “hallucinate” or provide misinformation, and the efforts needed to improve the technical abilities of present forms of AI should also be taught. Teachers and institutions should find ways to develop and enhance students’ critical thinking and analytical skills first, as these technologies are first introduced and refined for practice.

### Teaching Practical Skills in AI Use

At present, there is very little teaching on how to practically use AI tools for medical students and residents. This includes the use of these tools for personal, academic, and medical purposes. Perhaps the first step in medical education reform is to normalize the use of AI as a teaching and learning aid. AI has been shown, for example, to ameliorate teaching through helping with teaching concepts; creating and improving scenario modeling, courses, and content; and traditional curriculum and coursework [14]. Technologies that rely on large language models can help with developing curricula and teaching plans [40], generating teaching aids [41], simplifying complex medical concepts [42], and pretesting examination questions [43]. AI can also enhance self-directed learning for medical students. ChatGPT is being used by medical students to practice clinical scenarios, access medical literature, and study for examinations [44]. AI applications are currently being developed, for example, to improve case-based learning and decision-making skills [45,46]. In addition, they can analyze students’ responses in real time and provide immediate feedback and insights into students’ comprehension and learning progress [47]. As large language models become increasingly integrated, skills in prompt engineering and the development and refinement of appropriate inputs for generative AI are also needed to maximize the efficacy of these tools [48].

In addition to openness around the use of AI as a teaching and learning aid, training is needed on how to practically use AI tools as adjuncts to traditional clinical decision-making tools. The integration of AI into medical practice will require professionals to learn how to adapt workflows and communicate effectively with these tools. For example, AI scribes used to assist with documentation in patient encounters may require providers to learn to explicitly describe physical examination findings or to edit initial documentation effectively [49]. Surgical residents should be taught the use of AI tools used for surgical planning early in training [50]. Additionally, learners should be trained on how to communicate the process, risks, benefits, and alternatives of AI use to patients [34].

While it is encouraged that AI is used in medical education, safeguards will equally be necessary to address issues with academic integrity [14] while using ChatGPT and other such technologies for examinations, assignments, and assessments [44]. Guidelines encompassing accountability systems, ethical considerations, privacy, and moral and integrity issues can be used to help address academic integrity issues [40]. In addition, educating students on how to avoid plagiarism in conjunction with plagiarism detection and language analysis software can promote responsible use of these tools [51].

### Increasing Technological Aptitude

In addition to training medical learners on the use of existing AI technologies, a possible long-term goal to improve medical education on AI is to develop the technological aptitude. This includes skills in coding, Python language, mechanisms of data leakage, and an overview of how AI tools are developed [34]. The combination of clinical aptitude and understanding of the practical problems these tools need to solve make physicians uniquely positioned to assist with the production of these technologies. At present, however, many physicians lack the technical skills to help with AI development. Down the line, medical schools will need to consider how they can train physicians to do both.

## Conclusion

As AI tools become increasingly integrated into medical practice, they will offer powerful solutions to problems of uncertainty in medicine. These tools have the potential to address reducible forms of uncertainty, including the lack of available clinical information and scientific studies, limits to physician ability, and provider personal bias in decision-making. These tools may also improve the irreducible forms of uncertainty to some extent, increasing our ability to make case predictions from class probabilities and develop models of disease. Despite these capabilities, these tools will never be able to overcome foundational knowledge problems in medicine and pose ethical concerns that must be addressed. Nonetheless, AI tools are being used in practice, and trainees must learn the scope of these technologies, the ethical and legal challenges they pose, and how to use them practically. In the future, trainees should also be taught technical skills of how to develop these technologies. AI has reached medicine, and the medical profession is being asked time and time again to adapt; medical education reform is crucial in this transformation.

## Abbreviations

AI: artificial intelligence
CRP: C-reactive protein
GCA: giant cell arteritis
